# “But I Gathered My Courage”: HIV Self-Testing as a Pathway of Empowerment Among Ugandan Female Sex Workers

**DOI:** 10.1177/1049732320978392

**Published:** 2021-01-10

**Authors:** Jonas Wachinger, Daniel Kibuuka Musoke, Catherine E. Oldenburg, Till Bärnighausen, Katrina F. Ortblad, Shannon A. McMahon

**Affiliations:** 1 Heidelberg University, Heidelberg, Germany; 2 International Research Consortium, Kampala, Uganda; 3 University of California, San Francisco, California, USA; 4 Harvard T.H. Chan School of Public Health, Boston, Massachusetts, USA; 5 University of Washington, Seattle, Washington, USA; 6 Africa Health Research Institute, Durban, South Africa; 7 Johns Hopkins Bloomberg School of Public Health, Baltimore, Maryland, USA

**Keywords:** Uganda, East Africa, HIV self-testing, female sex worker, empowerment, resources, agency, achievements, qualitative, in-depth interviews

## Abstract

HIV self-testing (HIVST) increases HIV testing in diverse populations, but little is known about the experiences of individuals who self-test. We used a five-step framework approach to analyze 62 qualitative interviews with 33 female sex workers (FSWs) participating in an HIVST trial in urban Uganda. Notions of empowerment emerged from the data, and findings were interpreted based on Kabeer’s empowerment framework of resources, agency, and achievements. We found that access to HIVST bolstered empowerment because it increased participant’s time and money (resources), control of testing circumstances and status disclosure (agency), and sense of competency (achievements). In addition, we found that knowledge of HIV status empowered participants to better control HIV-related behaviors (agency) and recognize a new sense of self (achievements). This suggests that the availability of HIVST can facilitate feelings of empowerment, meriting a higher awareness for benefits outside of linkage to HIV treatment and prevention services.

## Introduction

HIV self-testing (HIVST) is becoming an alternative to facility-based HIV testing globally. It has been shown to increase recent and frequent HIV testing among those at high HIV risk (Johnson et al., 2017; Ortblad et al., 2017), but a gap in the literature remains on how the process of self-testing and learning one’s HIV status in private affects the individual. For individuals at high risk of HIV acquisition (such as female sex workers [FSWs] and their clients), frequent HIV testing is recommended to detect new infections (World Health Organization, 2015). HIV testing plays a central role for both HIV prevention and treatment (Joint United Nations Programme on HIV and AIDS [UNAIDS], 2014). Those who test negative can initiate pre-exposure prophylaxis (PrEP) for HIV prevention and engage in general behavior changes (e.g., condom use and partner selection) to prevent infection, whereas those who test positive can initiate HIV treatment that improves health outcomes and decreases further HIV transmission (especially if started soon after HIV infection; World Health Organization, 2014). HIV testing is thus a central part of reaching the UNAIDS’ (2014) 90–90–90 target for helping end the AIDS epidemic: that 90% of people living with HIV know their status, 90% of those initiate antiretroviral therapy (ART), and 90% of those achieve viral suppression.

In sub-Saharan Africa, the majority of HIV testing is performed in facilities (e.g. by health workers in health facilities; Wong et al., 2019; Obermeyer et al., 2014). This approach has numerous structural and individual-level barriers to uptake. Structural barriers include the financial burden associated with transportation to and payment for HIV testing (Tokar et al., 2018), limited facility hours (Wanyenze et al., 2017), and stigmatization by health workers (Ameyan et al., 2015; Chanda et al., 2017; Wanyenze et al., 2017). Individual-level barriers include misconceptions regarding HIV transmission and one’s own HIV risk (Wang et al., 2009), fear of receiving a positive HIV test result (Beattie et al., 2012; Chanda et al., 2017; King et al., 2017), and of having to face the stigma associated with an HIV infection (Chanda et al., 2017; Wanyenze et al., 2017; for a more extensive review, see Tokar et al., 2018). These barriers are often amplified for FSWs because they face stigmatization by the general population and are at a high risk of HIV infection (Tokar et al., 2018). Thus, new models for HIV testing that overcome structural and individual-level barriers to testing uptake are needed (Wong et al., 2019), especially among FSWs in high prevalence settings.

Oral HIVST—which tests for HIV antibodies in oral fluid and was first approved by the U.S. Food and Drug Administration in 2012 (Ingold et al., 2019)—allows individuals to test themselves and receive results within 30 minutes. This mode of HIV testing not only provides fast results (thus enhancing convenience) but also excludes the necessity of drawing blood (thus reducing physical pain; Figueroa et al., 2015). Oral HIV self-tests have proven to be highly sensitive and specific in different settings, including populations with high HIV risk in Sub-Saharan Africa (Piwowar-Manning et al., 2010; Zachary et al., 2012). However, studies also suggest that some individuals may have difficulties correctly executing HIVST and interpreting their results (e.g., Figueroa et al., 2018; Ortblad et al., 2018).

Although this new HIV testing technology has the potential to overcome barriers to facility-based testing, it also gives rise to several concerns. For example, because HIVST detaches HIV testing from counseling, there are concerns that it might cause psychological harm (including suicidal ideation) if individuals self-test HIV-positive (Wood et al., 2014). Additional concerns include potential violence or social harm by partners, family members, or others who might force or coerce someone to HIV “self”-test and discover their HIV status (Napierala Mavedzenge et al., 2013; World Health Organization, 2013; Youngs & Hooper, 2015).

In Sub-Saharan Africa, FSWs are among the populations at the highest risk of HIV infection (Baral et al., 2012; Vandepitte et al., 2011), and major barriers to facility-based testing, as outlined above, inhibit repeat testing (Tokar et al., 2018). Studies introducing HIVST to this population have shown that this technology increases recent and repeat HIV testing (Ortblad et al., 2017). These results align with research on HIVST uptake in other populations with a high risk of HIV infection and persistently low testing rates, both in Sub-Saharan Africa and globally (for a systematic review on the quantitative evidence for HIVST, see Johnson et al., 2017).

Fewer studies have explored the introduction of HIVST qualitatively to understand how individuals both utilize and experience HIVST. Central themes in available studies include appreciation of the privacy, convenience, ease of use, and painlessness associated with HIVST (Figueroa et al., 2015; Indravudh et al., 2017; Ngure et al., 2017; Okoboi et al., 2019; Pant Pai et al., 2013), and the manner in which individuals can use self-testing kits with—or distribute them to—(potential) sexual partners (Carballo-Diéguez et al., 2012; Maman et al., 2017).

Several studies have mentioned that participants felt “empowered” by their access to HIVST (e.g., Martinez et al., 2014; Ngure et al., 2017); however, little information is provided regarding how empowerment is understood and how it manifests in the individual’s HIVST experience. At the same time, although reported rarely in empirical studies, concerns regarding HIVST, such as forced testing (Scott, 2014), often relate to a potential disempowering effect on HIVST users. Youngs and Hooper (2015) in their analysis of the potential ethical implications of HIVST propose that it might empower users by allowing them to “take control over how they test for HIV,” and by “reducing the imbalance that exists between doctor and patient” in the context of facility-based testing (Youngs & Hooper, 2015, p. 810).

The suggested relationship between HIVST and (dis-)empowering experiences remains underexplored. In this study, we fill this gap in the literature by investigating how FSWs experience HIVST and how these experiences relate to notions of (dis-)empowerment.

## Method

### Theoretical Underpinning

Defining and measuring empowerment in the context of intervention delivery has been the subject of considerable debate across disciplines, leading to an increasing understanding of its multidimensionality and multidisciplinarity (Pratley, 2016). In this study, we follow Kabeer’s (1999) definition of empowerment as “the process by which those who have been denied the ability to make strategic life choices acquire such an ability” (p. 435). This definition resonates with a number of points that have become central to the empowerment discourse. First, empowerment is understood as a process, instead of an endpoint, acknowledging how it is both iterative (empowering experiences or developments can reshape goals or the ability to pursue them; Cattaneo & Chapman, 2010) and relative (empowerment does not happen in a vacuum, but depends on the previous status and the reference group; Mason, 2005). Second, empowerment is coined by the strategic life choices of the respective agents themselves, which acknowledges that empowerment has “occurred if it results from the agency of the person who feels empowered” (Narayan, 2005, p. 22), based on their own goals and values, instead of being granted to them. Third, the notion of acquiring an ability previously denied acknowledges how systematic inequalities between individuals and groups across different spheres (e.g., interindividual, economical, and political) are at the core of empowerment (Petesch et al., 2005).

Such systematic inequalities, and the process of overcoming them, shape the interactions between individuals, but also between the individual and formal or informal institutions (Narayan, 2005). In the context at hand, it is therefore essential to extend the scope beyond the effects of the process of HIVST on the individual Ugandan FSW, and also focus on how the availability of HIVST changes FSWs relations with others (e.g., their clients and peers), with formal and informal institutions (e.g., the health system), and the community as a whole.

In her work, Kabeer (1999) proposes a seminal framework that allows capturing empowerment across the dimensions of resources, agency, and achievements. Resources, per Kabeer, are the necessary preconditions for decision-making, not only in the material and economic sense but also in terms of human and social resources. Agency refers to the “ability to define one’s goals and act upon them” (Kabeer, 1999, p. 438), including what is often referred to as the “power within” or “sense of agency” to make decisions, bargain, deceive, or resist. Finally, achievements are often at the core of discussions about empowerment and refer to the outcomes of an intervention in terms of both physical and mental well-being (Kabeer, 1999).

In this study among Ugandan FSWs, we give voice to the participants themselves, and examine how HIVST influences their achievements, their (perceived) capacity to take action, and their relations with others. We therefore employ Kabeer’s multidimensional framework, which allows us to understand how the experiences around HIVST shaped participants’ perceptions.

### Study Setting

This study took place in Kampala, the capital city of Uganda, where ~13,000 FSWs work and one in three are living with HIV (Centers for Disease Control and Prevention, 2014). All FSWs in Kampala have access to free HIV testing and treatment services through Uganda’s Most at Risk Population Initiative (MARPI; Uganda Ministry of Health, 2020). At the time of this study, 2016 to 2017, oral HIVST was not available outside of research settings; the Uganda Ministry of Health launched oral HIVST in 2019 (Atukunda, 2019).

### Intervention Design

**Figure 1.**
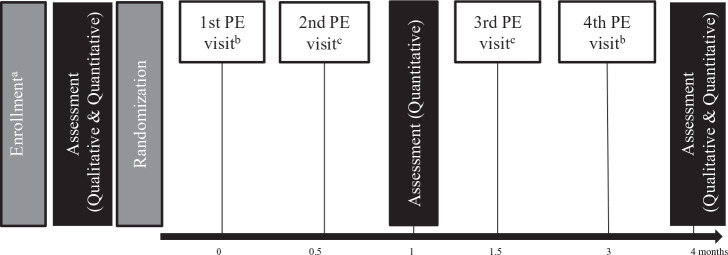
The design of the HIVST randomized trial participants engaged in. *Note.* HIVST = HIV self-testing; PE = peer educator; FSW = female sex worker. ^a^960 participants (≥18 years, reported exchanging sex for money or goods in the past month, were not knowingly living with HIV, had not recently tested for HIV [past 3 months], and had not previously used an oral HIV self-test) were recruited by PEs. PEs were selected based on their trust and respect within the FSW community and received a 2-day training on how to conduct and interpret self-tests and how to encourage further health-seeking behavior. We randomized 120 groups (eight participants and one PE) to one of the three study arms. ^b^PEs gave all participants condoms. In addition, participants in the direct provision arm received one oral HIV self-test; participants in the facility collection arm received a coupon for one self-test to be redeemed at participating clinics. ^c^PEs gave all participants condoms.

From October 2016 to March 2017, 960 participants were engaged in a randomized trial testing peer-based models of HIVST delivery among FSWs (ClinicalTrials.gov: NCT02846402): see Figure 1. In partnership with MARPI or Kampala-based nongovernmental organizations, we first selected 120 peer educators (PEs) who were well-respected in their communities. We trained these PEs on how to convey HIV prevention information and how to deliver HIV self-tests or encourage referrals for free HIV testing services (in the control arm only). Each PE then recruited eight eligible peers from their own network to participate in the study (*N* = 960 participants). Eligible peers had exchanged sex for money or goods in the past month, did not know their HIV status or previously tested HIV-negative over 3 months ago, and had not previously used an oral HIV self-test. All enrolled participants completed four PE visits at 0, 0.5, 1.5, and 3 months following enrollment, where they received information on HIV prevention. Participants randomized to one of the two intervention arms received either an HIV self-test or a coupon for one HIV self-test free of cost at Months 0 and 3. Participants in the intervention arms were trained by their PEs on how to perform and interpret a self-test, and to seek a confirmatory test at a health care facility if a self-test was positive. Participants received one self-test at a time to reduce the likelihood of tests being used on or sold to others.

This study was designed in collaboration with FSW stakeholders, including MARPI officials and NGO leaders, who shared their insights on how to best deliver a new technology to FSWs and gain trust within their community. The HIV self-test used in this study was the OraQuick Rapid HIV-1/2 Antibody Test (OraSure Technologies, Bethlehem, PA), which included a pictorial and written step-by-step instruction guide (available in both Luganda and English). Further details on the trial design and quantitative outcomes are published elsewhere (Ortblad et al., 2017).

### Data Collection and Sample Characteristics

We randomly selected 5% of participants in the two intervention arms to complete in-depth, semi-structured qualitative interviews, with the understanding that more participants would be sampled if saturation was not reached. Selected and willing participants each completed two interviews, one at baseline (before their first PE visit) and another after 4 months, following completion of all PE visits (here referred to as “follow-up interview”). The baseline interview focused on participants’ experiences in sex work, HIV testing and status disclosure, and HIVST opinions, including chances and concerns with this new testing technology. The follow-up interview focused on participants’ experience with HIVST (intervention arms only), as well as their life histories and relationship with clients and other FSWs. Trained qualitative researchers, all with a graduate-level education and prior qualitative research experience, conducted face-to-face, in-person interviews in either Luganda or English at a private location selected by participants. All interviews were audiotaped and later transcribed and translated into English.

In total, we completed 62 interviews with 33 participants. Twenty-nine participants completed both baseline and follow-up interviews, three participants did not complete the follow-up interview, and one participant was not available for the baseline interview and only completed the follow-up interview. The baseline interviews on average lasted 49 minutes (range: 18–84 minutes), the follow-up interviews on average lasted 47 minutes (range: 32–75 minutes). Participants had a median age of 30 years (range: 20–40 years), and 90% (*n* = 30) of participants reported completing at least primary education. At baseline, a majority of participants (*n* = 31) had previously tested for HIV at least once. At follow-up, all participants had tested for HIV over the course of the study, and the vast majority (*n* = 26) reported their last HIV test to be a self-test. Four participants reported self-testing HIV-positive over the course of the study. Participants had a median of five clients on an average working night (range: 3–12 clients) and earned substantially more for the provision of condomless vaginal sex (range: US$3–US$30) versus vaginal sex with a condom (range: US$0.90–US$15). Roughly half of participants at baseline reported inconsistent condom use with clients. For a detailed summary of participants’ demographic characteristics, see the table in the Supplemental files.

### Data Analysis

**Figure 2.**
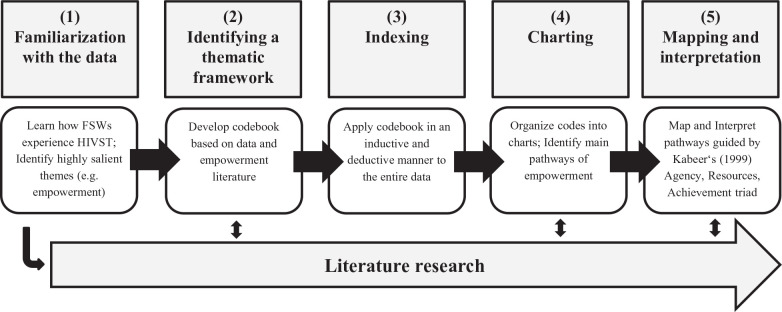
The five-step framework approach following Pope et al. (2000) used to analyze the qualitative data. *Note.* HIVST = HIV self-testing; FSW = female sex worker.

We analyzed the data following the 5-step framework approach (Pope et al., 2000): see Figure 2. First, co-authors immersed themselves in the data (Step 1). Then, JW and SM developed a codebook, began reviewing the existing literature (Step 2), and started to apply deductive and inductive coding to a set of 10 transcripts. Once a codebook was finalized, JW coded all transcripts (*N* = 62; Step 3). By including both the baseline and follow-up interviews, we could analyze participants’ attitudes about HIVST both before and after they had access to this new testing technology. As coding progressed, JW and SM had weekly debriefing calls to discuss emerging themes and mitigate personal biases. Over the course of charting the data (Step 4), JW, KO, and SM identified the main pathways of empowerment. To further tease out the underlying mechanisms of these pathways, we applied Kabeer’s (1999) framework to the charted data, mapping how each pathway manifests across the dimensions of resources, agency, and achievements. We used MAXQDA 12.3.6 (VERBI GmbH, Berlin, Germany) to facilitate analysis.

### Ethical Considerations

This study received ethical approval from the Institutional Review Board at the Harvard T.H. Chan School of Public Health (IRB16-0885) and Mildmay Uganda Research Ethics Committee (REF 0105-2016). All researchers were trained on ways to maintain confidentiality and use nondiscriminatory language when working with vulnerable populations (i.e., FSWs). We obtained written informed consent from all study participants

## Results

We identified two main pathways through which HIVST bolstered empowerment in the lives of study participants: (a) being able to test in private and at a time of one’s choice, and (b) acquiring knowledge of HIV status. The first pathway focuses on accessibility to and usage of HIVST, whereas the second pathway focuses on how learning one’s HIV status affects life and work realities. Although the latter pathway is not necessarily exclusive to HIVST, it is facilitated by the availability of HIVST, which has been shown to significantly increase recent and repeat HIV testing. We present both empowering and disempowering aspects of these pathways according to participants’ experiences. Table 1 summarizes our results following Kabeer’s empowerment framework of resources, agency, and achievements.

**Table 1.** FSW empowerment through access to HIVST and knowledge of HIV status

Dimensions of Empowerment^1^	Access to HIVST		Knowledge of HIV Status	
	*Positive Effect* ^2^	*Negative Effect*	*Positive Effect*	*Negative Effect*
**Resources** *Both material as* *well as human and* *social resources that* *enhance the ability* *to exercise choice* ^2^	Gain time, money, social support, bodily strength – *No distance/transport issues, no wait times* – *Can test clients who insist on condomless sex* – *Can build friendships via collective testing* – *No loss of blood or “life force” in saliva-based testing*	Lose care linkages *Less counseling; no screenings for other STIs*	Increased access to primary health care ^(+)3^ *Through establishing routine health seeking behavior after learning HIV-positive status* Gain money ^(+)^ *Less perceived need to refuse clients who insist on live sex*	Lose money *Foregoing clients due to desire to stay negative*^(-)^ */to reduce clients and ‘preserve life force’*^(+)^
**Agency** *The individual’s ability to define their own goals and act accordingly– including observable action, as well as meaning, motivation, and purpose* ^2^	Control testing circumstances *Timing, location, surroundings, how bodily samples and test results are used (who has access), who is tested (self or others)* Control result disclosure Avoid stigma *No discriminating gaze from health workers, other clients*	No control re: faulty test results *Due to misinterpretation or wrong execution*	Control re: own risks taken *FSWs can make informed decision what further risks to take; Potential violence by clients perceived as less threatening* New ability to exert power ^(+)^ *Agency to subject others to the risk of HIV infection; dependent on will to earn money, desire for revenge, moral values* Control re: the moment of HIV treatment initiation ^(+)^ *Ability to maintain health (and physical appearance) by starting early treatment*	Lose self-esteem, security, sense of control ^(+)^ *Loss of energy and hope*
**Achievements** *Outcomes in terms of well-being. In the context of this study also including outcomes that have a positive effect on the individual’s (mental) health*	Bolster one’s strength and ability *Feel prepared for facility-based testing* Feel valued and trusted by the health system *Feel capable and trusted with an important medical technology*	New problematic convictions re: HIV and testing *Re: test window, HIV transmission, confirmatory testing* New suspicions re: foreign medical technologies *Skepticism against tests not yet sanctioned by local government*^4^	Gain sense of self-efficacy and of ‘doing something right in life’ ^(-)^ *Encouragement for regular testing to preserve status and for reducing the provision of condomless sex increases with each negative result* Gain sense of self and feel reinvigorated to change life circumstances *A “wakeup call”; emboldened to change jobs, destructive family arrangements, to access treatment*^(+)^*/remain healthy*^(-)^	Feel unable to trust others *As there is now something precious to lose*^(-)^ *or out of fear of stigmatization*^(+)^ Feel forced to identify (and be identified) with either positive or negative peers

1 Definitions for resources, agency, and achievements according to Kabeer (1999)

2 Positive and negative in this context are understood in terms of empowering effects on participants, which do not necessarily translate into equally positive or negative results in terms of HIV prevention and treatment. Opposing effects are discussed in the discussion.

3 In those cases where the empowering effects of knowing one’s status are particular for either a seropositive or seronegative HIV test result, this is indicated with superscript (+) or (-) respectively

4 At time of data collection

### Empowerment Through Access to HIVST

#### Resources

##### Empowering effects

Participants reported several ways in which HIVST positively influenced the resources available to them. First, HIVST allowed gains in both time and money. Participants said that peer-delivered HIVST did not “tie you down for many hours” (age 30 years, follow-up interview) because it did not require traveling to or waiting at a health facility: a process that can often take a full day. Participants also described how HIVST might allow them to increase their financial resources by not only saving transport costs and clinic fees but also by enabling them “to test those customers who do not want to put on condoms” (age 32 years, baseline interview) and then providing this service, for which they often earn double.

Second, sharing the self-testing experience with peers was described as a moment of bonding, thus strengthening their social network. The fact that the self-test kits used oral fluid also influenced participant’s perceived resources, as many reported how both the pain of a needle, as well as the loss of blood connected with facility-based testing drains one’s own energy and saps the “blood sustaining my life” (age 28 years, follow-up interview).

##### Disempowering effects

As a drawback, participants described how HIVST disconnected them from the health facility and resources or services available there (e.g., counseling, testing for sexually transmitted infections). Participants discussed how a lack of counseling might lead to negative health effects, as “the self-test may shock you and you commit suicide or [you do] not follow up to get ARVs” (age 30 years, follow-up interview). However, these reported concerns were hypothetical; no one described experiencing such negative effects themselves.

#### Agency

##### Empowering effects

Participants reported that having access to HIVST allowed them to control their testing circumstances—especially in regard to time, place, and the presence of others—which was the central advantage of this “new technology” (age 29 years, follow-up interview). Participants also reported increased agency due to the ability to control who learns their HIV test results. Participants repeatedly explained how facility-based testing in most cases means that your status will be disclosed to everyone in the waiting area, as “your attitude changes, even your facial expression can be identified. [. . .] It is a public place; people get to know what has happened to you” (age 35 years, follow-up interview). They discussed how this involuntary HIV status disclosure can have serious consequences. If one tests HIV-positive, they must face the stigma associated with HIV, but if they test HIV-negative, peers might feel envious or affronted, resulting in social exclusion. One participant even reported attempts of deliberate HIV infection:

We keep it as a secret, you can’t just discuss it to your friends that you tested and found out that you were negative. Your friend can make you fall into a trap with a client who is HIV-positive. She can say “heeee,” she is boasting around [. . .]. You just need to keep quiet about your status. (age 28 years, follow-up interview)

Participants also discussed plans to test others, especially clients (both consensually and secretly), to increase financial resources and their own agency. Participants reported how clients were often unwilling to test for HIV and how they generally mistrusted HIV test results presented by clients because there are health workers who “don’t give true results. [. . .] At times you can get a client [who] bribes the nurses whereby he could be positive and they bring out negative results” (age 33 years, follow-up interview). Thus, they felt that the ability to test others for HIV gave them a sense of independence and control, including the ability to confirm a client’s HIV test result before choosing to engage in condomless sex.

##### Disempowering effects

Participants reported concerns that the self-test results might not be as accurate as the facility-based HIV test results; participants described insecurities about their ability to correctly perform the tests and interpret the results. “You fail to interpret the results and you may interpret it as a negative result but still you are not sure of the result,” thereby giving results that are “not genuine” (age 35 years, follow-up interview) or inconclusive. One participant, for example, did not correctly follow the instructions and received an inconclusive self-test result that left her unsure of what to do next. Instead of re-testing, she waited several months “for you guys [study staff] to come back so I can tell you about it since you are the experts on this self-test because I knew you were returning” (age 30 years, follow-up interview). These insecurities may negatively affect participants’ agency as they raise doubts about one’s ability to act and can negate the other potentially empowering effects of HIVST.

#### Achievements

##### Empowering effects

The experience of using an HIVST for many participants resulted in an increased belief in their own strength and ability. For example, the processes of “counseling oneself” (age 32 years, follow-up interview) largely based on what many participants had previously experienced amid HIV testing at health facilities, and “strengthening the heart” (age 27 years, follow-up interview) before performing an HIV self-test were reported as empowering experiences. One participant who had not tested for 6 years described how, after receiving her self-test, she “first stared at the kit for a while because I was so scared of what results the kit could bring. But I gathered my courage and took the test,” and that she “had finally gathered my strength” (age 37 years, follow-up interview). Participants also discussed how HIVST further prepared them for future self-testing or facility-based testing, which they previously avoided because of stories heard about or experiences made with coerced HIV testing.

Participants also discussed a preference for learning that they were living with HIV through HIVST instead of facility-based testing. One of the four participants in the qualitative sample who self-tested HIV-positive described how her experience was not easy, but made her stronger:

I first went and slept a bit. [After waking up] I cried a little, then afterwards I gained my confidence. [. . .] I first thought the result that it was showing might not be genuine, [. . .] but they had told us that in case we don’t agree with the result, we were given some clinics where we would go for a confirmatory test. So I went and they tested me again and the result was still the same. [. . .] That is when I had to agree with the outcome and I started planning to go and start getting treatment and I started taking it. (age 28 years, follow-up interview)

Another participant who self-tested HIV-positive said, “It is good I found the truth like I did [. . .] so all is well” (age 40 years, follow-up interview). For many participants, being trusted enough by the health system to perform HIVST, particularly as it wasn’t yet available to the general public, made them feel valued and sparked feelings of pride and strength among “us sex workers [who] know what we do” (age 35 years, follow-up interview).

##### Disempowering effects

The fact that HIVST was not yet sanctioned by the Ugandan government at the time of the study increased FSWs’ mistrust in those introducing the technology, including not only the study team but also the PEs, potentially contributing to group fragmentation. For example, participants reported concerns regarding Western medical hegemony, with one participant arguing that “those are things for white people and they come with their side effects” (age 32 years, follow-up interview). Similarly, the fact that a stigmatized group, such as FSWs, was chosen for piloting this new technology increased mistrust in some instances: “Let me ask you [. . .] why have you introduced this method first to the FSWs?” (age 32 years, baseline interview). One participant reported being too afraid to use the HIV self-test given to her: “I was hearing some rumors and so I got scared; that the HIV self-test kit is not yet legally in use, and some other people were saying that we are going to run mad after use” (age 30 years, follow-up interview). In addition, participants expressed confusion regarding the fact that HIV was now tested using oral fluid versus blood: “We have grown up being told that there is no HIV in saliva [. . .]. Trust me you are going to have a lot of trouble explaining yourselves to the masses so that they understand you” (age 40 years, follow-up interview).

### Empowerment Through Knowledge of HIV Status

#### Resources

##### Empowering effects

Regardless of personal status, several participants reported how knowledge of an HIV-positive status could—in certain domains—increase the resources available to them. For example, the frequent linkage to care after treatment initiation resulted in receiving much better HIV-unrelated, primary health care, especially for those who previously had avoided visiting health facilities. One participant explained how “I no longer feel cold all the time and I don’t fall sick all the time [. . .] because I am getting strong medicine” (age 28 years, follow-up interview).

For some, an HIV-positive status facilitated increased earnings. Participants reported how they knew peers who, following a positive test result, now saw no need to reject clients who insisted on condomless sex, for which FSWs earn around double compared with sex with a condom: “If I found out that I am positive, trust me I wouldn’t mind anymore whether the condom is on, or it bursts or whatever. I will leave that to the client” (age 29 years, follow-up interview). This ability to earn money with knowledge of an HIV-positive status was further increased by a culture of referrals, as some participants reported sending clients who insisted on “live sex” to FSWs known to be living with HIV and rumored to fulfill these demands. FSWs living with HIV were perceived as “rich compared to us [HIV-negative FSWs]” (age 28 years, follow-up interview).

##### Disempowering effects

At the same time, learning one’s status in some cases led to decreased earnings, and therefore to a reduction of available resources, regardless of the test result. For those who tested HIV-negative, participants reported that they no longer wanted to take risks, and therefore would have to forego clients. Some participants who tested HIV-positive reported an urge to take better care as to not risk reinfection and to preserve one’s health and “healthy looks” (age 38 years, follow-up interview).

#### Agency

##### Empowering effects

Due to financial pressure or sexual violence by clients, participants frequently experienced a lack of agency in the decision to engage in condomless sex with clients. Therefore, participants who tested HIV-positive reported that knowledge of their HIV status led to a shift in agency: Knowing that it was likely that condomless sex would be of less risk for themselves than it would be for their clients, they for the first time felt that they were in a position to decide to place a person who had power over them at risk. Although participants reported this agency to not always be translated into action, or, if so, be expresses in different ways (by, for example, changing the criteria based on which they would accept or reject clients, or whether or not to use condoms), it led to a perceived redefinition of the power structures in the FSW–client relationship.

Similarly, knowledge of an HIV-positive status gave participants an ability to make a decision often perceived as a moral one. On one hand, participants reported how they themselves or peers they knew would offer condomless sex to clients. This was not only reported as being a purely economic reasoning to increase earnings but also as a way to exact revenge on clients in general, as clients as a group were viewed as responsible for their infection: “So many people out there when they get to know that they are infected, they go on a rampage to infect others because they themselves were infected by someone” (age 31 years, follow-up interview). However, these comments were mainly hypothetical, and those participants who actually self-tested HIV-positive over the course of the study emphasized their intention to strictly use condoms with their clients:

[If a client does not want to use a condom] I let him go and he gets another sex worker. Some sex workers accept live sex. Others are like me; they don’t accept live sex though they know that they are sick. (age 24 years, follow-up interview)

On the other hand, some participants saw protecting others from an HIV infection as their responsibility or as a religious act of charity: “I do not want to kill you and then you too tomorrow go to infect someone else. [. . .] It’s not all about the money” (age 25 years, follow-up interview).

Knowledge of HIV-positive status also gave participants the agency to decide when to initiate treatment, which was seen as a way to maintain health, appearance, and the ability to care for others.

##### Disempowering effects

Those who tested HIV-positive also reported how the test result could have negative effects on their perceived agency. Although, for example, participants were more confident in their interactions with health care workers and the health system in general, they were not always able to translate changes in their perceived agency into actions when interacting with clients:

On that very day when I tested I wanted to get clients with condoms only, but still I didn’t get them. I felt I wanted to change but I couldn’t do it. . . . I ended the day when I got two clients who wanted live sex. (age 30 years, follow-up interview)

Furthermore, knowledge of an HIV-positive status in some cases reduced energy and hope, leading to an at least temporary inability to make life choices (age 28 years, follow-up interview).

#### Achievements

##### Empowering effects

Knowledge of an HIV-negative status, especially after avoiding HIV testing for a long time and living with the perception that “at the end of the day everyone is going to get HIV” (age 25 years, follow-up interview), was described by participants as drastically increasing one’s own well-being. One participant described how, for the first time in a long time, she had the feeling of “doing something right and I have to continue doing so” (age 31 years, follow-up interview), and for others the empowering effect of an HIV-negative test result continued to increase with each self-test taken.

Regardless of whether the result received was positive or negative, participants reported how they gained a new sense of “how your life is”, as “getting to know your status makes you feel free” (age 35 years, baseline interview). For several participants, the test result was a reason to push for a change in life circumstances, ranging from quitting sex work to leaving the country, in addition to a general perception that “my risk has gone down ever since I took the test” (age 37 years, follow-up interview).

##### Disempowering effects

Simultaneously, knowing one’s HIV status was reported as forcing one to identify with one of two groups (HIV-positive or HIV-negative) within the FSW community, resulting in potential rejection from the respective other. In addition, some participants struggled to trust people around them, ranging from clients to peers and partners, out of fear of infection (if they received a negative test result) or stigmatization (if they received a positive test result).

## Discussion

From this study, two pathways of empowerment via HIVST emerged among FSWs: empowerment via HIV testing in private and at a time of one’s choice, and empowerment via knowledge of HIV status. Both pathways manifested across the three dimensions of Kabeer’s (1999) empowerment framework: resources, agency, and achievements. With regard to the process of HIVST, participants described testing as empowering because it was convenient, status disclosure was mitigated, and being entrusted with a test was evidence of one’s own capability (in the eyes of oneself and the broader heath system). With regard to the acquired knowledge about HIV status, participants described how such knowledge facilitated access to social and medical resources, underpinned a sense of achievement, and provided a motivation to modify some HIV risk-related behaviors. Although negative consequences of HIVST, such as intimate partner violence or suicidal thoughts, were not reported by participants when narrating their own experiences, a number of disempowering facets emerged, such as potential loss of financial and social resources and uneven health care linkages.

The influence of HIVST on FSWs’ perceived agency found in this study echoes work from previous qualitative studies on FSWs and other key populations in Sub-Saharan Africa. Maman and colleagues (2017) described how both FSWs and their clients expressed enthusiasm regarding HIVST, especially regarding its privacy, and how women demonstrated agency regarding how and to whom to offer self-testing kits and how to avoid conflict (Maman et al., 2017). Similarly, FSWs participating in a qualitative study on the potential of HIVST in Raika, Uganda (Burke et al., 2017), and PrEP users in Kenya (Ngure et al., 2017) appreciated the confidentiality and convenience of HIVST. For people living in informal settlements in South Africa, the agency to decide when and where to test and the resulting possibility to gain independence from mistrusted health workers and facilities was key in reaching individuals who declined facility-based testing (Martínez Pérez et al., 2016). In this study, we expand on this knowledge by showing the meaning this newfound agency assumes in the lives of Ugandan FSWs, including how the ability to avoid discrimination by health workers and to reduce the risks of unintended status disclosure resulted in a feeling of security and control.

HIVST can shift HIV testing itself into a private realm, but facilitated linkage to health facilities is needed for counseling, confirmatory testing, and treatment initiation. Several studies across settings have addressed issues of consent to testing, or reported that individuals feel forced to test for HIV when visiting health facilities for other reasons (Groves et al., 2010; Martínez Pérez et al., 2016; Obermeyer et al., 2014). Our study echoes these findings. In addition, FSWs reported skepticism regarding test results (whether their own or those of friends or clients) stemming from the ability to bribe health workers to deliver HIV-negative results. The finding that FSWs lost these concerns after testing for HIV at home suggests that the availability of HIVST might increase trust in health facilities—or at least make it easier to contend with unpleasant experiences there. This links to a broader discussion on how to reduce barriers for marginalized populations to visit health facilities—not only in the context of HIV testing and treatment, but also for general primary care. It is possible that this effect might increase once the reliability of HIVST is established in the public perception. Although participants only reported resorting to facility-based testing to confirm the result they had received through HIVST, a reverse process where HIVST is used to establish the trustworthiness of a facility is plausible, too. While we are not aware of studies testing this assumption in the context of self-testing, patients’ trust in their surgeon, for example, increased when a second opinion was sought and confirmed the suggestion made by the initial medical professional (Axelrod & Goold, 2000). In a similar way, HIVST might assume the role of a second opinion in contexts where individuals do not yet have a trusted health facility for accessing health services, both HIV-related and general.

At the same time, participant’s perceptions of self-testing allowing them to avoid health care facilities might have negative consequences for their own health over time (e.g., screenings for other sexually transmitted infections [STIs], general health checkups). This and the possibility mentioned by some participants that self-testing clients and using the result to decide whether or not to have unprotected sex can lead to a neglect of other STIs and associated health risks, emphasizes the importance of comprehensive educational programs and future research on the effects of the introduction of HIVST on general health-seeking behavior.

Learning one’s HIV status, whether positive or negative, can be an empowering process, but can also require a fundamental reshaping of one’s social identity. In a population at high HIV risk (such as FSWs in Sub-Saharan Africa), a majority will still test HIV-negative, despite subjective assumptions to the contrary (Ortblad et al., 2019). In the present study, participants expressed fatalism regarding the inevitability of an HIV infection during the baseline interviews, but also surprise and reassurance following a (unexpected) negative test result at follow-up. While these empowering aspects apply to learning one’s *negative* HIV status, testing HIV-*positive* was also connected with its own positive effects on empowerment. In this respect, studies have shown that knowledge of HIV-positive status can facilitate access to certain resources (Updegraff et al., 2002), including informal social networks mediating the disruptions caused by the diagnosis (Ciambrone, 2002). This is in line with findings showing how learning one’s HIV status, often after being afraid of testing for an extended period of time, can have a positive effect on mental health, regardless of whether the test result is negative or positive (Ortblad et al., 2020). The way in which some participants framed an HIV-positive test result as plausible (and often suspected) and voiced concrete plans and goals for their lives as a person living with HIV further resembles ways to integrate the diagnosis into a coherent narrative of one’s life and towards ‘living positively’ (Levy & Storeng, 2007). This also mirrors research indicating that an incorporation of HIV into one’s own identity often begins with the diagnosis, but can also start when individuals suspect their own HIV-positive status (Baumgartner, 2007).

Informal social support structures, including among peers, can mitigate sexual risks for FSWs (Choudhury et al., 2015). In our study, it emerged how knowledge of HIV status—especially as a member of a population at elevated HIV risk—can force individuals to identify or align with one of two groups (those who are or are not living with HIV), which in turn can restrict social networks and make it much harder to contend with the realities of sex work and daily risk of HIV infection. This social dimension of knowledge of HIV status underscores how a test result holds meaning not only in terms of how an FSW views herself, but also how she relates to those in her immediate community. This is further exemplified by the concerns voiced by some participants that being known as HIV-negative among peers can lead to social exclusion or even attempts of deliberate infection. Although the stigmatization of being HIV-positive has received considerable attention (e.g., Rankin et al., 2005), we are not aware of research regarding the stigmatization of HIV-negative FSWs in contexts of high HIV prevalence. With increasing HIV status knowledge being a central goal of HIV prevention efforts globally, this is an important area of future research.

In several cases, factors that may empower individuals may present negative consequences for others. This was the case when participants described their desire to test others, especially clients, secretly or against their will if needed. Similarly, empowerment of the individual is not necessarily equally beneficial in terms of a broader public health perspective. This was the case when FSWs reported an increase in resources and agency of peers after they tested HIV-positive, as they now felt they were able to have unprotected sex with their clients. Although those participants who actually self-tested positive reported continued or increased insistence on protected sex, and the analysis of the quantitative data has shown no increase in sexual risk-taking behavior following an HIV-positive test result (Ortblad et al., 2019), this finding merits further attention. While intentionally spreading HIV to clients was only reported hypothetically in this study, it is echoed elsewhere; a study among FSWs in China found that up to 9% of FSWs considered the possibility of spreading HIV to take revenge (Lau et al., 2011), while clients of FSWs in India reported similar attitudes (Karandikar & Gezinski, 2012).

In general, our analysis of empowering and disempowering effects is based on participants’ own accounts and their experiences of using HIVST, but also their perception of future, personal benefits. Certain empowering effects mentioned by participants can seem unlikely (e.g., clients who have refused testing or refused to share their test results later agreeing to use an HIVST before engaging in unprotected sex) or exaggerated (e.g., complete independence from stigmatizing health workers). Some of these effects were mentioned by participants before receiving their first self-testing kit whereas others again were hypothetical in nature. For example, participants might have overestimated the amount of self-tests they would perform, both on themselves and with clients, based on their experience of receiving self-tests free of cost. Additional research would inform the extent to which some of these effects persist or manifest now that the HIVST has been made widely available in Uganda.

After the conclusion of this trial, research among FSWs in Kenya showed that FSWs demonstrate considerable agency in both distributing self-tests to sexual partners and reducing the risk of resulting conflict (Maman et al., 2017). This evidence mitigates some concerns about intimate partner violence, but further research on how the broad availability of self-tests shapes FSW–client relationships would be important.

Certain changes reported—or hoped for—by participants occurred differently in different relationships. For example, participants often reported experiencing changes in their interactions with health workers or the broader health system after learning their status through HIVST. At the same time, participants reported several instances where they themselves had experienced a change in their perceived agency in the relationship with their clients (e.g., insisting on protected sex after learning one’s HIV-negative status), which proved impossible to effectively enact. This reflects how changes in empowerment often do not occur equally across different spaces and interpersonal relationships.

In some instances, participants’ knowledge of HIV and HIVST highlighted messaging that merits emphasis in future sensitization, particularly on the point that HIVST can only show an infection after a 3-month delay. As outlined in the introduction, a number of studies have reported individuals’ difficulties to correctly perform and interpret HIV self-tests, despite training and pictorial step-by-step instructions (for a systematic review of the reliability of HIVST as compared to testing performed by health workers, see Figueroa et al., 2018). However, knowledge is central to empowerment; individuals, for example, have to know their legal rights to draw on them (e.g., Deshmukh-Ranadive, 2005), and general education is a central part of the empowerment discourse (e.g., Rowlands, 1997). In the context at hand, factually correct knowledge (e.g., of how to conduct and correctly interpret tests, of one’s own HIV status, and about HIV in general) is one deciding factor in whether or not the empowering mechanisms outlined throughout this article are expressed. The way in which knowledge across facets can be increased in the context of HIVST should be considered by both interventions and research following its public availability.

This study has limitations. First, questions on empowerment were not included in the structured qualitative guides. Empowerment only emerged as a central concept during analysis. It is therefore possible that some notions of empowerment over the course of the study were not reported in interviews. Second, data collection concluded shortly after participants in the respective study arms received their second HIVST kit or voucher. It is therefore difficult to infer longer-term effects regarding self-test use and the degree that empowering effects persist. Third, at the time of this study (2016–2017), HIVST was not yet available to the general public in Uganda. With the 2019 endorsement of HIV self-tests by the Ugandan Ministry of Health (Atukunda, 2019) and self-tests being now available to the public for around US$3 per test (Bulterys et al., 2020), certain effects on empowerment might have changed, for example, FSWs’ mistrust in those providing an “illegal” technology to a marginalized group and excitement surrounding access to a “new technology.” Finally, FSWs are vastly different within and across countries in terms of, for example, HIV prevalence, work realities, access to information and care, or the legal situation (Baral et al., 2012; Shannon et al., 2018); broad comparisons merit caution.

HIVST has been shown to improve HIV testing rates in a variety of settings and could therefore be of great value in reaching the UNAID’s 90–90–90 goal. This study has shown that among Ugandan FSWs, HIVST also has a broad variety of empowering effects across each dimension of Kabeer’s empowerment triad, and therefore contributes to understanding the positive effects HIVST may have on both more comprehensive HIV testing among key populations, as well as on the individual using it. However, this study also highlighted how empowerment of individuals may conflict with general public health interests. This exemplifies how empowerment is an important and still understudied concept in relation to global health interventions. The topic emerged as highly salient in relation to this work, but the difficulty of gauging how individual empowerment can come at the expense of collective public health may merit more pointed analysis. We further urge that future public health interventions seeking to enhance empowerment consider the broad spectrum of pathways through which programs may empower or disempower individuals.

## Supplemental Material

sj-pdf-1-qhr-10.1177_1049732320978392 – Supplemental material for “But I Gathered My Courage”: HIV Self-Testing as a Pathway of Empowerment Among Ugandan Female Sex Workers Supplemental material, sj-pdf-1-qhr-10.1177_1049732320978392 for “But I Gathered My Courage”: HIV Self-Testing as a Pathway of Empowerment Among Ugandan Female Sex Workers by Jonas Wachinger, Daniel Kibuuka Musoke, Catherine E. Oldenburg, Till Bärnighausen, Katrina F. Ortblad and Shannon A. McMahon in Qualitative Health Research

## References

[bibr1-1049732320978392] Ameyan W. Jeffery C. Negash K. Biruk E. Taegtmeyer M. (2015). Attracting female sex workers to HIV testing and counselling in Ethiopia: A qualitative study with sex workers in Addis Ababa. African Journal of AIDS Research, 14(2), 137–144. 10.2989/16085906.2015.1040809 26223330

[bibr2-1049732320978392] Atukunda N. (2019). Government launches oral HIV self-test kit. https://www.monitor.co.ug/News/National/Government-launches-oral-HIV-self-test-kit/688334-5290032-14g0vst/index.html

[bibr3-1049732320978392] Axelrod D. A. Goold S. D. (2000). Maintaining trust in the surgeon-patient relationship: Challenges for the new millennium. Archives of Surgery, 135(1), 55–61. 10.1001/archsurg.135.1.55 10636348

[bibr4-1049732320978392] Baral S. Beyrer C. Muessig K. Poteat T. Wirtz A. L. Decker M. R. . . . Kerrigan D. (2012). Burden of HIV among female sex workers in low-income and middle-income countries: A systematic review and meta-analysis. The Lancet Infectious Diseases, 12(7), 538–549. 10.1016/S1473-3099(12)70066-X 22424777

[bibr5-1049732320978392] Baumgartner L. M. (2007). The incorporation of the HIV/AIDS identity into the self over time. Qualitative Health Research, 17(7), 919–931. 10.1177/1049732307305881 17724104

[bibr6-1049732320978392] Beattie T. S. H. Bhattacharjee P. Suresh M. Isac S. Ramesh B. M. Moses S. (2012). Personal, interpersonal and structural challenges to accessing HIV testing, treatment and care services among female sex workers, men who have sex with men and transgenders in Karnataka state, South India. Journal of Epidemiology and Community Health, 66(Suppl. 2), 42–48. 10.1136/jech-2011-200475 22495772

[bibr7-1049732320978392] Bulterys M. A. Mujugira A. Nakyanzi A. Nampala M. Taasi G. Celum C. Sharma M. (2020). Costs of providing HIV self-test kits to pregnant women living with HIV for secondary distribution to male partners in Uganda. Diagnostics, 10(5), 318. 10.3390/diagnostics10050318 32438594 PMC7277977

[bibr8-1049732320978392] Burke V. M. Nakyanjo N. Ddaaki W. Payne C. Hutchinson N. Wawer M. J. . . . Kennedy C. E. (2017). HIV self-testing values and preferences among sex workers, fishermen, and mainland community members in Rakai, Uganda: A qualitative study. PLOS ONE, 12(8), e0183280. 10.1371/journal.pone.0183280 28813527 PMC5558930

[bibr9-1049732320978392] Carballo-Diéguez A. Frasca T. Balan I. Ibitoye M. Dolezal C. (2012). Use of a rapid HIV home test prevents HIV exposure in a high risk sample of men who have sex with men. AIDS and Behavior, 16(7), 1753–1760. 10.1007/s10461-012-0274-2 22893194 PMC3458207

[bibr10-1049732320978392] Cattaneo L. B. Chapman A. R. (2010). The process of empowerment: A model for use in research and practice. The American Psychologist, 65(7), 646–659. 10.1037/a0018854 20873882

[bibr11-1049732320978392] Centers for Disease Control and Prevention. (2014). Crane survey: Select results from recent surveys, Kampala, 2012/3. http://fileserver.idpc.net/library/Crane-survey_Uganda.pdf

[bibr12-1049732320978392] Chanda M. M. Perez-Brumer A. G. Ortblad K. F. Mwale M. Chongo S. Kamungoma N. . . . Oldenburg C. E. (2017). Barriers and facilitators to HIV testing among Zambian female sex workers in three transit hubs. AIDS Patient Care and STDs, 31(7), 290–296. 10.1089/apc.2017.0016 28581820 PMC5512327

[bibr13-1049732320978392] Choudhury S. M. Toller Erausquin J. Park K. Anglade D. (2015). Social support and sexual risk among establishment-based female sex workers in Tijuana. Qualitative Health Research, 25(8), 1056–1068. 10.1177/1049732315587282 25991735

[bibr14-1049732320978392] Ciambrone D. (2002). Informal networks among women with HIV/AIDS: Present support and future prospects. Qualitative Health Research, 12(7), 876–896. 10.1177/104973202129120331 12214676

[bibr15-1049732320978392] Deshmukh-Ranadive J. (2005). Gender, power, and empowerment: An analysis of household and family dynamics. In Narayan D. (Ed.), Measuring empowerment: Cross disciplinary perspectives (pp. 103–121). The World Bank.

[bibr16-1049732320978392] Figueroa C. Johnson C. Ford N. Sands A. Dalal S. Meurant R. . . . Baggaley R. (2018). Reliability of HIV rapid diagnostic tests for self-testing compared with testing by health-care workers: A systematic review and meta-analysis. The Lancet HIV, 5(6), e277–e290. 10.1016/S2352-3018(18)30044-4 29703707 PMC5986793

[bibr17-1049732320978392] Figueroa C. Johnson C. Verster A. Baggaley R. (2015). Attitudes and acceptability on HIV self-testing among key populations: A literature review. AIDS and Behavior, 19(11), 1949–1965. 10.1007/s10461-015-1097-8 26054390 PMC4598350

[bibr18-1049732320978392] Groves A. K. Maman S. Msomi S. Makhanya N. Moodley D. (2010). The complexity of consent: Women’s experiences testing for HIV at an antenatal clinic in Durban, South Africa. AIDS Care, 22(5), 538–544. 10.1080/09540120903311508 20229376 PMC2992088

[bibr19-1049732320978392] Indravudh P. P. Sibanda E. L. d’Elbée M. Kumwenda M. K. Ringwald B. Maringwa G. . . . Taegtmeyer M. (2017). “I will choose when to test, where I want to test”: Investigating young people’s preferences for HIV self-testing in Malawi and Zimbabwe. AIDS, 31(Suppl. 3), S203–S212. 10.1097/QAD.0000000000001516 28665878 PMC5497773

[bibr20-1049732320978392] Ingold H. Mwerinde O. Ross A. L. Leach R. Corbett E. L. Hatzold K. . . . Baggaley R. C. (2019). The Self-Testing AfRica (STAR) Initiative: Accelerating global access and scale-up of HIV self-testing. Journal of the International AIDS Society, 22(Suppl. 1), e25249. 10.1002/jia2.25249 30907517 PMC6432103

[bibr21-1049732320978392] Johnson C. C. Kennedy C. Fonner V. Siegfried N. Figueroa C. Dalal S. . . . Baggaley R. (2017). Examining the effects of HIV self-testing compared to standard HIV testing services: A systematic review and meta-analysis. Journal of the International AIDS Society, 20(1), 21594. 10.7448/IAS.20.1.21594 28530049 PMC5515051

[bibr22-1049732320978392] Joint United Nations Programme on HIV and AIDS. (2014). 90-90-90: An ambitious treatment target to help end the AIDS epidemic. http://files.unaids.org/en/media/unaids/contentassets/documents/unaidspublication/2014/90-90-90_en.pdf

[bibr23-1049732320978392] Kabeer N. (1999). Resources, agency, achievements: Reflections on the measurement of women’s empowerment. Development and Change, 30(3), 435–464. 10.1111/1467-7660.00125

[bibr24-1049732320978392] Karandikar S. Gezinski L. B. (2012). “These girls gave me AIDS. Why should I use condoms?” Clients of sex workers in Kamathipura express their attitudes about HIV. Journal of HIV/AIDS & Social Services, 11(2), 140–151. 10.1080/15381501.2012.678123

[bibr25-1049732320978392] King E. J. Maman S. Dudina V. I. Moracco K. E. Bowling J. M. (2017). Motivators and barriers to HIV testing among street-based female sex workers in St. Petersburg, Russia. Global Public Health, 12(7), 876–891. 10.1080/17441692.2015.1124905 26707862 PMC6173944

[bibr26-1049732320978392] Lau J. T. F. Gu J. Tsui H. Chen H. Wang R. Hu X. (2011). How likely are HIV-positive female sex workers in China to transmit HIV to others? Sexual Health, 8(3), 399–406. 10.1071/SH10106 21851782

[bibr27-1049732320978392] Levy J. M. Storeng K. T. (2007). Living positively: Narrative strategies of women living with HIV in Cape Town, South Africa. Anthropology & Medicine, 14(1), 55–68. 10.1080/13648470601106343 26873800

[bibr28-1049732320978392] Maman S. Murray K. R. Napierala Mavedzenge S. Oluoch L. Sijenje F. Agot K. Thirumurthy H. (2017). A qualitative study of secondary distribution of HIV self-test kits by female sex workers in Kenya. PLOS ONE, 12(3), e0174629. 10.1371/journal.pone.0174629 28346527 PMC5367822

[bibr29-1049732320978392] Martinez O. Carballo-Diéguez A. Ibitoye M. Frasca T. Brown W. Balan I. (2014). Anticipated and Actual Reactions to Receiving HIV Positive Results Through Self-Testing Among Gay and Bisexual Men. AIDS and behavior, 18(12), 2485–2495. 10.1007/s10461-014-0790-3 24858480 PMC4229402

[bibr30-1049732320978392] Martínez Pérez G. Cox V. Ellman T. Moore A. Patten G. Shroufi A. . . . Ibeto M. (2016). “I know that I do have HIV but nobody saw me”: Oral HIV self-testing in an informal settlement in South Africa. PLOS ONE, 11(4), e0152653. 10.1371/journal.pone.0152653 27044006 PMC4820175

[bibr31-1049732320978392] Mason K. O. (2005). Measuring women’s empowerment: Learning from cross-national research. In Narayan D. (Ed.), Measuring empowerment: Cross disciplinary perspectives (pp. 89–102). The World Bank.

[bibr32-1049732320978392] Napierala Mavedzenge S. Baggaley R. Corbett E. L. (2013). A review of self-testing for HIV: Research and policy priorities in a new era of HIV prevention. Clinical Infectious Diseases: An Official Publication of the Infectious Diseases Society of America, 57(1), 126–138. 10.1093/cid/cit156 23487385 PMC3669524

[bibr33-1049732320978392] Narayan D. (2005). Conceptual framework and methodological challenges. In Narayan D. (Ed.), Measuring empowerment: Cross disciplinary perspectives (pp. 3–38). The World Bank.

[bibr34-1049732320978392] Ngure K. Heffron R. Mugo N. Thomson K. A. Irungu E. Njuguna N. . . . Baeten J. M. (2017). Feasibility and acceptability of HIV self-testing among pre-exposure prophylaxis users in Kenya. Journal of the International AIDS Society, 20(1), 21234. 10.7448/IAS.20.1.21234 28362073 PMC5467615

[bibr35-1049732320978392] Obermeyer C. M. Verhulst C. Asmar K. (2014). Could you have said no? A mixed-methods investigation of consent to HIV tests in four African countries. Journal of the International AIDS Society, 17, 18898. 10.7448/IAS.17.1.18898 24647205 PMC3959275

[bibr36-1049732320978392] Okoboi S. Twimukye A. Lazarus O. Castelnuovo B. Agaba C. Immaculate M. . . . King R. (2019). Acceptability, perceived reliability and challenges associated with distributing HIV self-test kits to young MSM in Uganda: A qualitative study. Journal of the International AIDS Society, 22(3), e25269. 10.1002/jia2.25269 30932364 PMC6441924

[bibr37-1049732320978392] Ortblad K. F. Kibuuka Musoke D. Ngabirano T. Nakitende A. Magoola J. Kayiira P. . . . Bärnighausen T. (2017). Direct provision versus facility collection of HIV self-tests among female sex workers in Uganda: A cluster-randomized controlled health systems trial. PLOS Medicine, 14(11), e1002458. 10.1371/journal.pmed.1002458 29182634 PMC5705079

[bibr38-1049732320978392] Ortblad K. F. Musoke D. K. Chanda M. M. Ngabirano T. Velloza J. Haberer J. E. . . . Bärnighausen T. (2020). Knowledge of HIV status is associated with a decrease in the severity of depressive symptoms among female sex workers in Uganda and Zambia. Journal of Acquired Immune Deficiency Syndromes, 83(1), 37–46. 10.1097/QAI.0000000000002224 31633611 PMC6898780

[bibr39-1049732320978392] Ortblad K. F. Musoke D. K. Ngabirano T. Nakitende A. Haberer J. E. McConnell M. . . . Oldenburg C. E. (2018). Female sex workers often incorrectly interpret HIV self-test results in Uganda. Journal of Acquired Immune Deficiency Syndromes, 79(1), e42–e45. 10.1097/QAI.0000000000001765 29847478 PMC6095458

[bibr40-1049732320978392] Ortblad K. F. Musoke D. K. Ngabirano T. Salomon J. A. Haberer J. E. McConnell M. . . . Bärnighausen T. (2019). Is knowledge of HIV status associated with sexual behaviours? A fixed effects analysis of a female sex worker cohort in urban Uganda. Journal of the International AIDS Society, 22(7), e25336. 10.1002/jia2.25336 31287625 PMC6615530

[bibr41-1049732320978392] Pant Pai N. Sharma J. Shivkumar S. Pillay S. Vadnais C. Joseph L. . . . Peeling R. W. (2013). Supervised and unsupervised self-testing for HIV in high- and low-risk populations: A systematic review. PLOS Medicine, 10(4), e1001414. 10.1371/journal.pmed.1001414 23565066 PMC3614510

[bibr42-1049732320978392] Petesch P. Smulovitz C. Walton M. (2005). Evaluating empowerment: A framework with cases from Latin America. In Narayan D. (Ed.), Measuring empowerment: Cross Disciplinary perspectives (pp. 39–67). The World Bank.

[bibr43-1049732320978392] Piwowar-Manning E. M. Tustin N. B. Sikateyo P. Kamwendo D. Chipungu C. Maharaj R. . . . Brooks Jackson J. (2010). Validation of rapid HIV antibody tests in 5 African countries. Journal of the International Association of Physicians in AIDS Care (Chicago), 9(3), 170–172. 10.1177/1545109710368151 PMC2989535 20530471

[bibr44-1049732320978392] Pope C. Ziebland S. Mays N. (2000). Analysing qualitative data. British Medical Journal, 320, 114–116. 10.1136/bmj.320.7227.114 10625273 PMC1117368

[bibr45-1049732320978392] Pratley P. (2016). Associations between quantitative measures of women’s empowerment and access to care and health status for mothers and their children: A systematic review of evidence from the developing world. Social Science & Medicine, 169, 119–131. 10.1016/j.socscimed.2016.08.001 27716549

[bibr46-1049732320978392] Rankin W. W. Brennan S. Schell E. Laviwa J. Rankin S. H. (2005). The stigma of being HIV-positive in Africa. PLOS Medicine, 2(8), e247. 10.1371/journal.pmed.0020247 16008508 PMC1176240

[bibr47-1049732320978392] Rowlands J. (1997). Questioning empowerment: Working with women in Honduras. Oxford: Oxfam. http://policy-practice.oxfam.org.uk/publications/questioning-empowerment-working-with-women-in-honduras-121185

[bibr48-1049732320978392] Scott P. A. (2014). Unsupervised self-testing as part public health screening for HIV in resource-poor environments: Some ethical considerations. AIDS and Behavior, 18(Suppl. 4), S438–S444. 10.1007/s10461-014-0833-9 24974124

[bibr49-1049732320978392] Shannon K. Crago A.-L. Baral S. D. Bekker L.-G. Kerrigan D. Decker M. R. . . . Beyrer C. (2018). The global response and unmet actions for HIV and sex workers. The Lancet, 392(10148), 698–710. 10.1016/S0140-6736(18)31439-9 PMC6384122 30037733

[bibr50-1049732320978392] Tokar A. Broerse J. E. W. Blanchard J. Roura M. (2018). HIV testing and counseling among female sex workers: A systematic literature review. AIDS and Behavior, 22(8), 2435–2457. 10.1007/s10461-018-2043-3 29464430 PMC6097720

[bibr51-1049732320978392] Uganda Ministry of Health. (2020). Most at Risk Population Initiative (MARPI). http://www.marpi.org/about-us

[bibr52-1049732320978392] Updegraff J. A. Taylor S. E. Kemeny M. E. Wyatt G. E. (2002). Positive and negative effects of HIV infection in women with low socioeconomic resources. Personality and Social Psychology Bulletin, 28(3), 382–394. 10.1177/0146167202286009

[bibr53-1049732320978392] Vandepitte J. Bukenya J. Weiss H. A. Nakubulwa S. Francis S. C. Hughes P. . . . Grosskurth H. (2011). HIV and other sexually transmitted infections in a cohort of women involved in high-risk sexual behavior in Kampala, Uganda. Sexually Transmitted Diseases, 38(4), 316–323. 23330152 PMC3920055

[bibr54-1049732320978392] Wang Y. Li B. Zheng J. Sengupta S. Emrick C. B. Cohen M. S. Henderson G. E. (2009). Factors related to female sex workers’ willingness to utilize VCT service: A qualitative study in Jinan city, northern China. AIDS and Behavior, 13(5), 866–872. 10.1007/s10461-008-9446-5 18770027 PMC2930910

[bibr55-1049732320978392] Wanyenze R. K. Musinguzi G. Kiguli J. Nuwaha F. Mujisha G. Musinguzi J. . . . Matovu J. K. B. (2017). “When they know that you are a sex worker, you will be the last person to be treated”: Perceptions and experiences of female sex workers in accessing HIV services in Uganda. BMC International Health and Human Rights, 17(1), 11. 10.1186/s12914-017-0119-1 28476153 PMC5420144

[bibr56-1049732320978392] Wong V. Jenkins E. Ford N. Ingold H. (2019). To thine own test be true: HIV self-testing and the global reach for the undiagnosed. Journal of the International AIDS Society, 22(Suppl. 1), e25256. 10.1002/jia2.25256 30912306 PMC6433601

[bibr57-1049732320978392] Wood B. Ballenger C. Stekler J. (2014). Arguments for and against HIV self-testing. HIV/AIDS: Research and Palliative Care, 6, 117–126. 10.2147/HIV.S49083 25114592 PMC4126574

[bibr58-1049732320978392] World Health Organization. (2013). Report on the first international symposium on self-testing for HIV: The legal, ethical, gender, human rights and public health implications of HIV self-testing scale-up. https://apps.who.int/iris/bitstream/handle/10665/85267/9789241505628_eng.pdf?sequence=1

[bibr59-1049732320978392] World Health Organization. (2014). Consolidated guidelines on HIV prevention, diagnosis, treatment and care for key populations. https://apps.who.int/iris/bitstream/handle/10665/128048/9789241507431_eng.pdf?sequence=1 25996019

[bibr60-1049732320978392] World Health Organization. (2015). Consolidated guidelines on HIV testing services: 5Cs: Consent, confidentiality, counselling, correct results and connection. https://apps.who.int/iris/bitstream/handle/10665/179870/9789241508926_eng.pdf?sequence=1&isAllowed=y 26378328

[bibr61-1049732320978392] Youngs J. Hooper C. (2015). Ethical implications of HIV self-testing. Journal of Medical Ethics, 41(10), 809–813. 10.1136/medethics-2014-102599 26276789

[bibr62-1049732320978392] Zachary D. Mwenge L. Muyoyeta M. Shanaube K. Schaap A. Bond V. . . . Ayles H. (2012). Field comparison of OraQuick ADVANCE Rapid HIV-1/2 antibody test and two blood-based rapid HIV antibody tests in Zambia. BMC Infectious Diseases, 12, Article 183. 10.1186/1471-2334-12-183 PMC3475053 22871032

