# Supplementary material for: “But I Gathered My Courage”: HIV Self-Testing as a Pathway of Empowerment Among Ugandan Female Sex Workers
Source: Qual Health Res. 2021 Jan 10;31(3):443–57. doi: 10.1177/1049732320978392 (PMC7887607; doi:10.1177/1049732320978392)
Supplement: sj-pdf-1-qhr-10.1177_1049732320978392 – Supplemental material for “But I Gathered My Courage”: HIV Self-Testing as a Pathway of Empowerment Among Ugandan Female Sex Workers [file sj-pdf-1-qhr-10.1177_1049732320978392.pdf]

Participant characteristics

Measured at baseline (n=33)	
Age (median, range)	30 (20 to 40)
Age when starting sex work (median, range)	21 (13 to 37)
Education (n)	
No formal	3
Primary/Junior	14
Secondary	13
Vocational	1
Tertiary	2
Monthly income, USD[1] (n)	
< $35	9
$35 - $75	4
$75 - $150	15
> $150	5
Timing of last HIV test (n)	
0-3 months	0
>3-6 months	14
>6-12 months	7
>12 months	10
Never tested	2
Participants' estimation of HIV prevalence	
Out of 10 FSW, estimated HIV-positive (median, range)	6.5 (3 to 9)
Out of 10 clients, estimated HIV-positive (median, range)	7 (2 to 10)
Number of clients/average night (median, range)	5 (3 to 12)
Inconsistent condom use with clients[2] (n)	17
Price for vaginal sex, USD[1] (median, range)	
With a condom	$3.00 ($0.90 to $15)
Without a condom	$6.00 ($3 to $30)
Measured at follow-up (n=32, as one participant was lost to follow-up)	
Tested for HIV since the start of the study	32
Used an HIV self-test	26
Self-tested HIV-positive over the course of the study	4

[1]Price categories in US dollars (USD); October 10th, 2016 exchange rate (1 USD = 3363.85 Ugandan Shillings).

[2]Defined as not using a condom with at least one client on an average working night
